# An Infodemic of Misinformation on Stem Cell Therapy Among the Population of Saudi Arabia: A Cross-Sectional Study

**DOI:** 10.3389/fmed.2022.789695

**Published:** 2022-03-02

**Authors:** Doaa Aboalola, Heba Badraiq, Rawiah Alsiary, Samer Zakri, Neda Aboulola, Loay Haneef, Dalal Malibari, Moayad Baadhaim, Khaled Alsayegh

**Affiliations:** ^1^King Abdullah International Medical Research Center, Jeddah, Saudi Arabia; ^2^King Saud bin Abdulaziz University for Health Sciences, Jeddah, Saudi Arabia; ^3^Ministry of National Guard Health Affairs, Jeddah, Saudi Arabia; ^4^King AbdulAziz University Hospital, Jeddah, Saudi Arabia

**Keywords:** misleading stem cell treatment information, false advertisement, unproven stem cell treatments, public awareness, public health, health care providers, knowledge, Saudi Arabia

## Abstract

In recent years, the industry of unproven stem cell-based therapies has been on the rise around the globe, putting patients at great risk of potential harm. In this cross-sectional study, we aimed to assess the level of knowledge and awareness of the general public, including patients and/or their relatives, in Saudi Arabia on stem cell therapy and to assess the degree of willingness to try stem cell-based treatment options, should it be offered to them.

**Methods:**

A voluntary questionnaire of 16 questions was distributed randomly through social media outlets.

**Results:**

In the survey of this study, 2,030 individuals participated. A total of 1,292 (63.6%) stated that they would accept stem cell therapy or would recommend it to their friends and relatives. Alarmingly, 72.1% of participants were unaware that using unapproved stem cell-based treatments may lead to serious health complications including cancer. More than 20% believed that stem cell therapy is already approved for organ/tissue regeneration. Worryingly, 60.6% of the physicians and 56.4% of the medical students stated that they would recommend stem cell treatment for their patients.

**Conclusions:**

There is a concerning spread of misinformation among the Saudi population, including physicians, regarding stem cell therapy. This calls for a targeted effort to raise awareness about the current status of stem cell treatment in the general public and among health care practitioners.

## Introduction

Clinics providing unproven stem cell-based interventions are widespread, offering patients stem cells or stem cell-derived products without sufficient scientific or clinical evidence which could put patients at great risk. (1). Businesses and clinics providing such treatments are flourishing in many countries around the world, and Saudi Arabia is not an exception (2). In recent years, there has been an alarming growth in its domestic market with clinics and beauty salons offering stem cell-based products and treatments (2). The publics' desire and willingness to try stem cell-based interventions, especially for chronic and debilitating diseases, is understandable. Therefore, this unprecedented spread of such clinics poses an immediate risk to all patients, especially when such unproven therapies could lead to detrimental health complications like tumor formation, blood clots, and blindness (3, 4).

For these reasons, it has become an absolute necessity for governments to regulate the operations of the aforementioned clinics to protect patients from harm and exploitation. In addition, raising public awareness by providing evidence-based educational platforms became important to counteract the danger posed by the spread of such unproven therapies and misinformation. Patients looking for information about stem cell-based interventions will usually resort to the internet to search, and they could fall prey to inaccurate conflicting information and claims found in advertisements, media, and blogs (2, 5, 6).

The stem cell field has become increasingly mystified by the desire to advance stem cell-based interventions to the clinic without adequate scientific support. Untested stem cell therapies are often described as “trials”, although the patient has to pay for the experimental treatment, which raises a complex of ethical, social, and economic concerns (7). Therefore, there is a need to improve the evaluation of stem cell therapies to differentiate between clinical trials supported by scientific rationale and trials without preclinical safety that might endanger patients and jeopardize the future of regenerative medicine (7).

Although the field of stem cells in both research and clinical applications has rapidly progressed globally, accurate knowledge about its current status among the public is still lacking, as multiple reports have shown (8–11). Alarmingly, this inadequate awareness could also be found among health care providers, as one study conducted in Italy has shown that two-thirds of the participating physicians did not have comprehensive knowledge about stem cells and their current approved use in therapy (12).

In our study, we aimed to assess the level of knowledge, awareness, and acceptance of the general public (including patients and their relatives) regarding stem cell use in Saudi Arabia and gave general recommendations to help raise the community's awareness and reduce the unnecessary harm of stem cell misuse. This is to move forward from our previous study focusing on cosmetics with “stem cells” (2) and shift the gear toward stem cell tourism aimed at making false claims for stem cell treatment for chronic illnesses.

## Materials and Methods

Ethical approval was obtained from the local research ethics committee (IRB) at King Abdullah International Medical Research Center (IRBC/0878/20). A voluntary questionnaire-based study of 16 questions using (Google form, USA) was distributed randomly to the public using social media platforms. Each participant agreed to participate by accepting the informed consent form before completing the questionnaire.

Raw data were collected and processed for any imprecisions or loss of information before the statistical analysis. All variables were assessed using (GraphPad Prism 8, San Diego, CA, USA), an analysis software, and results were reported as counts and percentages.

## Results

The results of this survey came from the general population which may represent a bias, as not all general public use social media; however, this issue may be considered minimum due to the high number of responses.

In this survey, a total of 2,030 individuals participated, with almost half of them being patients or related to patients (54.6%) in the Kingdom of Saudi Arabia (Table 1). Consistent with our previous study (2), the majority of participants (62.7%) were females, suggesting that women may have a higher propensity to engage with stem cell-related topics, which may be indicative of a higher interest compared to males in the Kingdom (Table 1). Most of the participants were between 25 and 50 years old (73.8%), and the education group with the highest participation was the bachelor's degree group (56%), followed by master's degree (22.1%), Ph.D. (8.1%), high school education (7.2%), diploma level education (6.1%), and lastly by 0.5% who did not complete high school (Table 1).

**Table 1:** Demographic characteristics of participants.

**Demographic characteristics**	**Group**	**Total = 2,030, (%)**
Gender	Female	1,272, (62.7 %)
	Male	758, (37.3 %)
Age (years)	18–24	199, (9.8 %)
	25–34	756, (37.2 %)
	35–50	743, (36.6 %)
	Above 50	332, (16.4 %)
Education	Below high school	12, (0.5 %)
	High school	146, (7.2 %)
	Diploma	123, (6.1 %)
	Bachelor	1,137, (56 %)
	Master's	448, (22.1 %)
	PhD	164, (8.1%)
Do you or anyone related to you suffer from a life-threatening illness?	Yes	1,084, (54.6 %)
	No	653, (31 %)
	Maybe	293, (14.4 %)

As we previously reported (2), the majority of participants (79.4%) lacked basic knowledge of the origins and types of stem cells and their use in medical applications (Supplementary Figure S1A). However, approximately 70.5% of the participants (*n* = 1,432) reported that they have a general understanding of stem cells (Supplementary Figure S1B). Nevertheless, this basic understanding was not attained by attending educational courses provided by accredited institutions, as 89.2% of the subjects stated that they had never received any form of education about the topic (Supplementary Figure S1C). Their knowledge was acquired by general education, recommendations by physicians, social media, family and friends, television and radio, and commercial companies (data not shown). It is worth noting that in our previous survey (2), we showed that 13% of the individuals who said they had stem cell knowledge gave wrong answers when asked basic stem cell-related questions.

**Figure 1:**
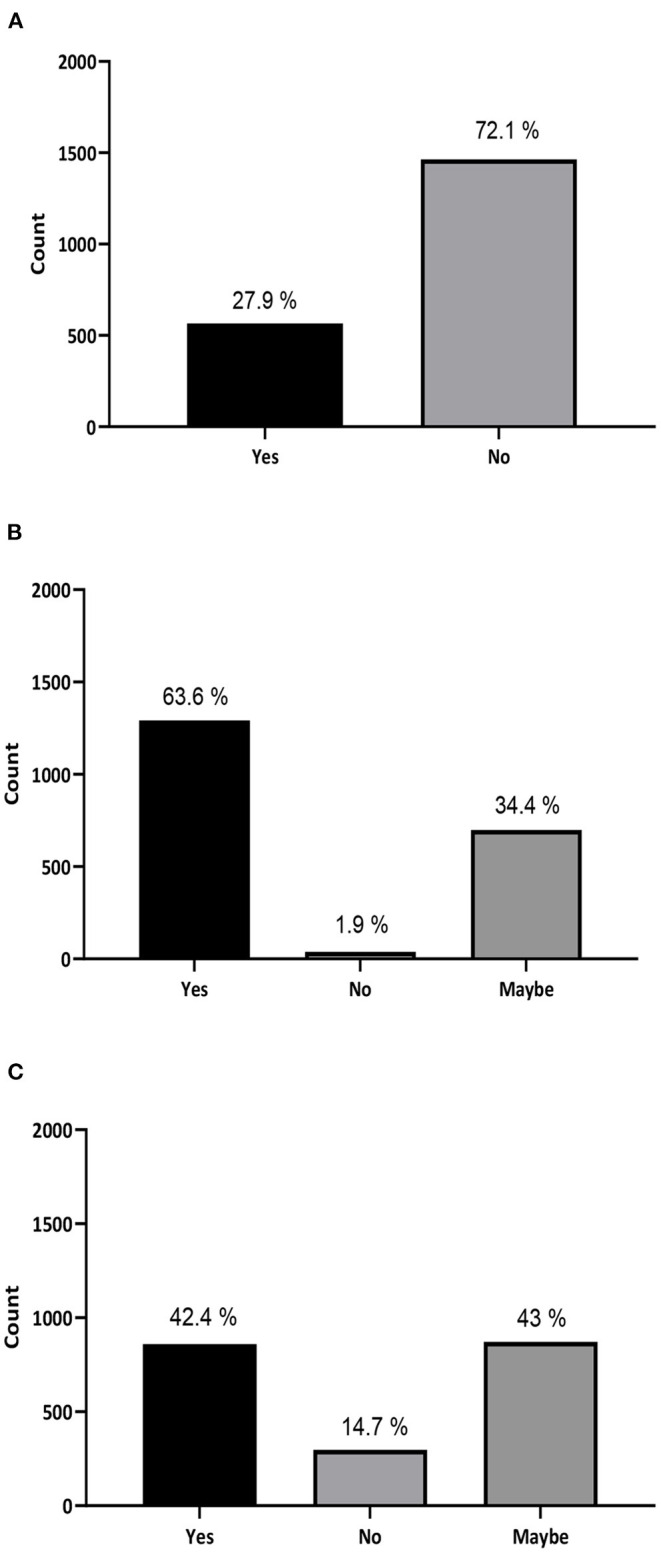
General attitude, acceptance, and knowledge toward stem cell treatment in the Kingdom of Saudi Arabia. **(A)** Do you know that using unapproved stem cell treatment may cause serious complications and may result in having cancer and/or other health complications? **(B)** If you or anyone related to you have a life-threatening illness, would you accept a stem cell treatment offered to you or recommend it? **(C)** Are you willing to travel and try a stem cell treatment or recommend it to a family member or friend?

About 72.1% of the subjects were not aware that using unapproved stem cell treatment may cause serious health complications (Figure 1A). In addition, a significant number of participants (438, 21.6%) think that stem cells are an approved treatment for hair and facials. Furthermore, 412 individuals (20.3%) believe that stem cells are currently in use for regenerating damaged organs. Among them, 14.2% trust that it is used to treat cancer and 4.9% think it is a cure for diabetes (data not shown) A total of 1,235 out of 2,030 (41.9%) were misinformed participants.

**Figure 2:**
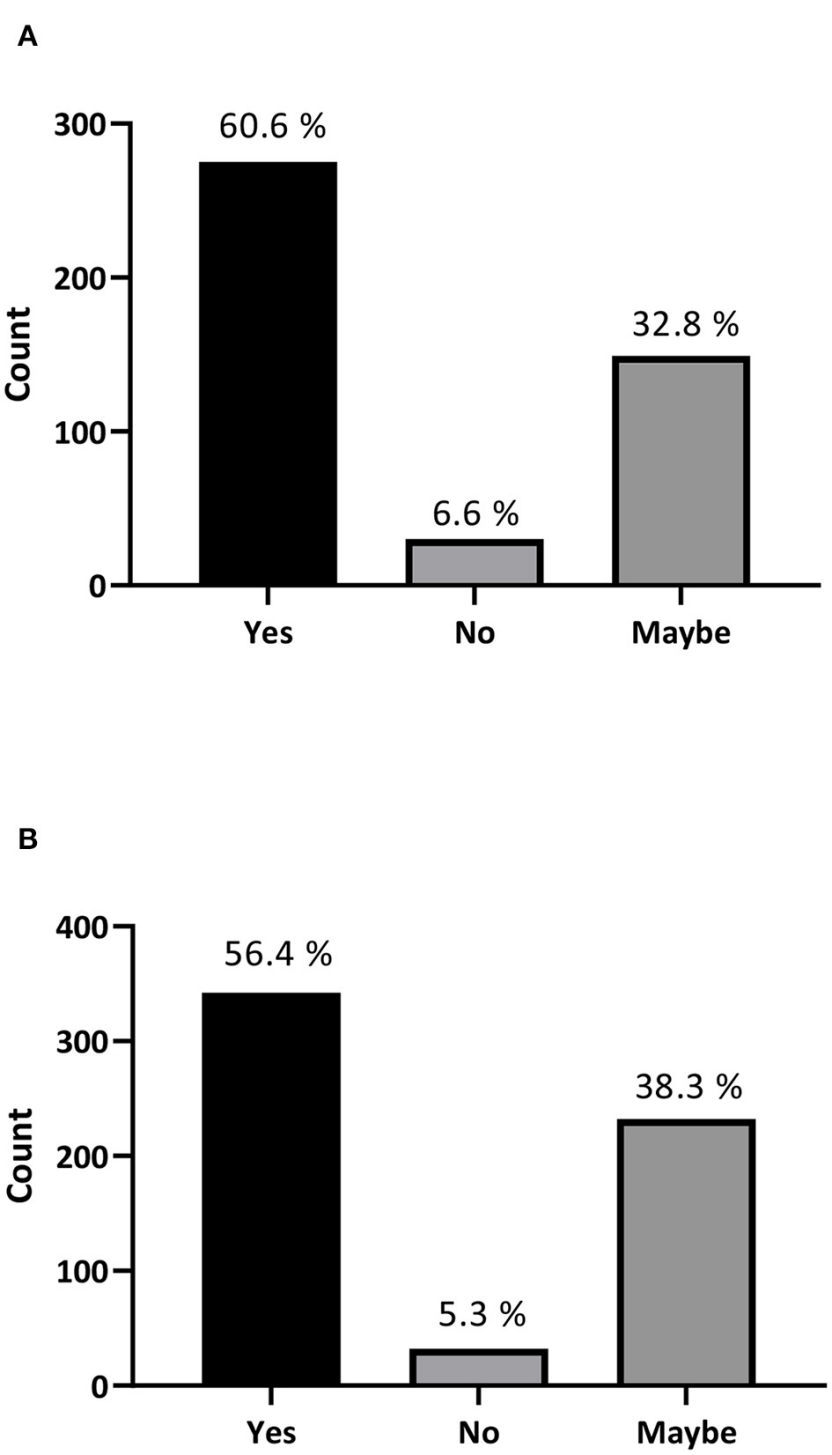
Stem cell treatment recommendations by physicians and medical students. **(A)** If you are a physician, would you recommend a stem cell treatment to your patients? **(B)** If you are a medical student, would you recommend a stem cell treatment?

Moreover, 63.6% of participants stated that they would accept stem cell treatment if offered to them or would recommend it to someone they know (Figure 1B), and 85.4% are willing or considered to travel abroad to try stem cell treatment or recommend traveling for treatment to a family member or a friend (Figure 1C). Surprisingly, our data revealed that 60.6% of the participating physicians (275 out of 454) and 56.4% of medical students (342 out of 606) would recommend stem cell treatment for their patients (Figures 2A,B). Interestingly, more than 90% of the participants stated that they were interested in developing their knowledge about stem cells (Supplementary Figure S2).

## Discussion

The main objective of this study was to investigate the level of knowledge and awareness among the Saudi Arabian population including health care providers on the usage of stem cells as a treatment option. In this report, a total of 2,030 participants were surveyed. The questionnaire included 16 questions to assess the opinion of participants about the usage of stem cells as a curable tool for treating serious illnesses. Female engagement in our study was twice as much as male engagement (62.7 and 37.3%, respectively), which could be indicative of an increased interest in the female group to learn more about stem cells and their potential in human health enhancement. This could also be a result of the increased volume of marketing campaigns carried out by cosmetic clinics around the Kingdom that specifically target women and market their products as containing stem cell-derived ingredients. The majority of our participants (73.8%) were aged between 25 and 50 years old, which is the age range that would most likely be the target of such marketing campaigns.

According to the survey results, the vast majority of participants (70.5%) considered themselves to have a general understanding of the topic of stem cells, however (79.4%) claimed to lack detailed knowledge. Yet more than 63% of participants answered with “Yes” when asked if they would be willing to try stem cell therapy if offered to them or their family members, with 34% answering with “Maybe”. Around 43% stated that they would be willing to travel abroad to get such treatment. With the spread of clinics providing unproven stem cell therapies around the globe, these numbers are more concerning than ever. They indicate an alarming propensity of Saudi patients and their families to try such unproven therapies.

Moreover, when asked about what approved stem cell treatments they know, around 20% of respondents stated that stem cell therapy is already approved for organ regeneration and 21.3% thought it is also a treatment option for cosmetic purposes for hair and facial skin. This could explain the acceptance we observed to try stem cell therapy and the willingness to travel abroad for it.

Almost 90% of the survey respondents stated that they have never attended any stem cell meetings or courses, which is consistent with a previously published report (13). Around 28% of people who claimed to have a detailed knowledge of stem cells stated that the source of their information was education, followed by health care clinics (25.3%).

The level of education of more than 86% of participants was a bachelor's degree or higher, which indicates that the current general education material is not a sufficient source to educate the public about the risks of unproven stem cell therapies and that additional educational efforts need to be implemented to raise awareness and protect patients and their families from uncontrolled medical care. It is also essential to provide health care professionals with accurate information about stem cells and their current therapeutic uses, as most people trust their health care providers as being a valuable and reliable source of information (9). This will eventually improve the decision-making power of patients regarding the use of stem cells as a novel and innovative method of treatment (13).

Around 19% of participants who claimed to have detailed knowledge about stem cells, stated that their source of knowledge was “social media”. This percentage is worrying because the abundance of inaccurate information on social media could give the patient a false sense of confidence which could lead them to make erroneous detrimental decisions regarding their ongoing health condition. Additionally, 15.3% of participants reported that they have heard about stem cells as a treatment option through family members or friends, and around 6% of participants mentioned that they have used commercial companies as a source of information. The majority of these commercial companies provide inaccurate information about their products and claim that it contains stem cells or ingredients derived from stem cells (2). This highlights the importance of close monitoring of such clinics and commercial companies and the products they offer by regulatory governmental agencies.

Moreover, unfortunately, more than 70% of the participants in this study were not fully aware of the serious possible consequences of using unapproved stem cells as a treatment. The public's limited amount of knowledge about stem cells and their approved treatments may render patients and their families vulnerable to falling prey to exploitation and may end up trying what is still at best, an experimental therapy, which in turn may overburden the Saudi health care system.

With the advent of stem cell research in the past two decades, the International Society for Stem Cell Research (ISSCR) was founded. General guidelines have been developed for the clinical translation of stem cell-based interventions by the ISSCR (14). The guidelines highlighted the need for rigorous scientific and clinical evidence during stem cell-based product development to ultimately provide safe and effective cell therapies to patients in need. However, as was observed in our present study, there is a significant willingness among patients (or their families) to pursue therapies that may lack a supporting body of evidence on safety or efficacy.

The ISSCR's Guidelines for Stem Cell Research and Clinical Translation, from 2006 to date, nurture conditions in which patients, researchers, industry, and stakeholders are confident that their interests will be advanced and protected when they collaborate in stem cell research (15, 16).

One of the major principles advocated by the ISSCR that should be implemented worldwide is transparency and availability of information to the general public (14). Researchers should always report the results of preclinical research irrespective of whether they are positive, negative, or inconclusive. In addition, there is a continuous ongoing policy to encourage regulatory bodies and stem cell agencies around the world to enforce regulations against the use of unapproved stem cell treatments (14, 16). To further counteract that spread of clinics that provide such treatments, the ISSCR has developed a section on their website where the public can report the activities of such disreputable clinics (17).

In Saudi Arabia, there are two public centers for cord blood banking that were subjected to a Fatwa (Islamic legal ruling) issued by the Muslim World League's Jurisprudential Council (10, 18). The first cord blood public bank was created at King Faisal Specialist Hospital and Research Center in 2006, followed by a second bank established in 2009 at King Abdullah International Medical Research Center (KAIMRC) (10). The KAIMRC Umbilical Cord Blood Bank is a non-profit public bank that provides hematological stem cells for patients in need and research purposes (9, 10, 19). Notably, the National Guard Health Affairs established The Saudi Stem Cell Donor Registry (SSCDR) in 2011, which is the first stem cell donor registry in the Middle East aimed to increase matching rates for patients (10, 20, 21). However, there is a general lack of knowledge among the Saudi population about the availability of such reliable centers and the services they provide (9). A more recent survey conducted by Almaeen et al. in 2021, has also shown that up to two-thirds of participants were unaware of these centers (22).

## Conclusions

**Figure 3:**
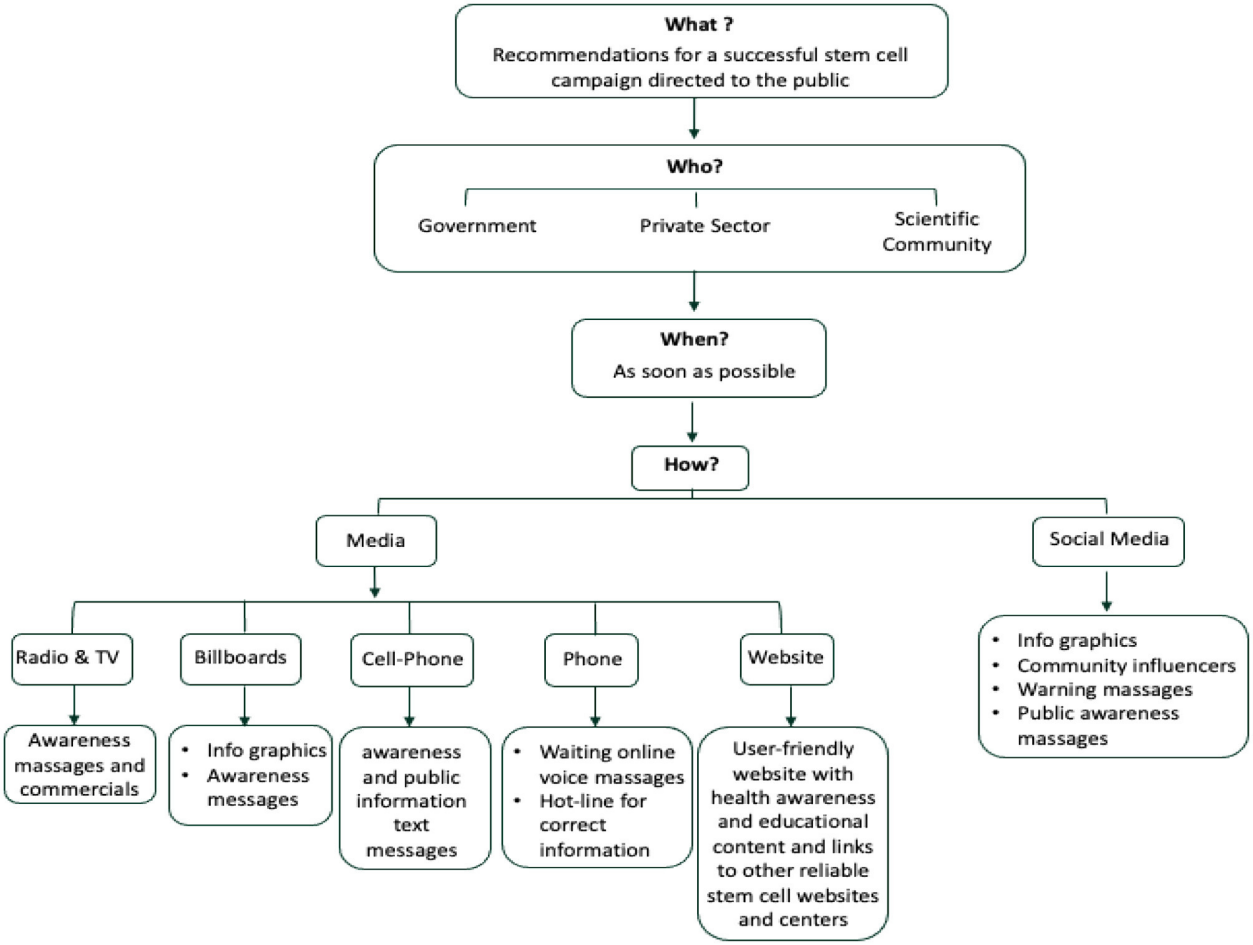
Diagram for the recommendations for a successful public stem cell campaign.

Our study has exposed an alarming propensity and willingness among the Saudi population to try stem cell therapies and it demonstrated a worrying lack of knowledge and awareness of the possible negative health consequences of such unproven treatments. It highlights an urgent need for educational campaigns that would provide the public with updated information about the current status of the stem cell research field. Similar to the successful “Quit Smoking” campaign by The Saudi Ministry of Health, we suggest launching a campaign that addresses this issue and helps decrease the gap of un-approved stem cell uses and dangers that are rapidly expanding with time. Finally, to help raise the awareness of the public and counteract the risky unethical stem cell misuse, the diagram in Figure 3 shows the recommendations for a successful campaign by answering four key questions: What, When, Who, and How (Figure 3). In addition, such campaigns should extend to include and engage health care professionals as they are considered a dependable and trustworthy source of information by patients and their families.

## Data Availability Statement

The original contributions presented in the study are included in the article/Supplementary Material, further inquiries can be directed to the corresponding authors.

## Ethics Statement

Ethical approval was obtained from the local research Ethics Committee (IRB) at King Abdullah International Medical Research Center (IRBC/0878/20). The patients/participants provided their written informed consent to participate in this study.

## Author Contributions

DA and NA completed raw data collection and processing. DA performed the data analysis. DA and KA wrote and edited the manuscript with input from HB, RA, SZ, NA, and LH. All authors had input in writing and finalizing the survey questions, contributed equally to distributing the survey, and approved the final manuscript.

## Conflict of Interest

The authors declare that the research was conducted in the absence of any commercial or financial relationships that could be construed as a potential conflict of interest.

## Publisher's Note

All claims expressed in this article are solely those of the authors and do not necessarily represent those of their affiliated organizations, or those of the publisher, the editors and the reviewers. Any product that may be evaluated in this article, or claim that may be made by its manufacturer, is not guaranteed or endorsed by the publisher.
