# Supplementary material for: An Infodemic of Misinformation on Stem Cell Therapy Among the Population of Saudi Arabia: A Cross-Sectional Study
Source: Front Med (Lausanne). 2022 Mar 2;9:789695. doi: 10.3389/fmed.2022.789695 (PMC8924302; doi:10.3389/fmed.2022.789695)
Supplement: Supplementary file 1 [file Data_Sheet_1.PDF]

JED-20-427780-71795

Assessment on Stem Cell Research Knowledge for Patients and their Relatives in the Kingdom of Saudi Arabia

King Abdullah International Medical Research Center
KAIMRC
APPROVED
IRB
Institutional Review Board

1. **Are you sick or anyone related to you sick or died due to a terminal illness?**
   - Yes
   - No

2. **Please specify the type of disease** ______________________

3. **How old are you?**
   - 18 - 24
   - 25-34
   - 35-50
   - Above 50

4. **Gender:**
   - Male
   - Female

5. **Education:**
   - Did not complete high school
   - High school
   - Diploma
   - Bachelor
   - Master's
   - PhD

6. **Do you have a general knowledge of stem cells?**
   - Yes
   - No
   - I do not know

7. **Do you have a detailed knowledge of stem cells?**
   - Yes
   - No
   - I do not know

8. **If your answer is yes for questions 6 or 7, what is your source of information about stem cells?**
   - Commercial companies
   - Social media
   - Education
   - Media (TV, Radio, etc.)
   - Clinic / Physician
   - Family / Friend

9. From what you have heard, Stem Cells are approved and used as a treatment in the clinic for:

- Cancer treatment
- Regenerating new organs
- Blood related genetic disorders and cancer
- Hair and Facial treatments
- Diabetes
- I do not know

10. Did you ever attend meetings regarding stem cells?

- Yes
- No

11. If you are a physician, would you recommend a stem cell treatment to your patients?

- Yes
- No
- I do not know
- Not Applicable

12. If you are a medical student, would you recommend a stem cell treatment?

- Yes
- No
- I do not know
- Not Applicable

13. If you or anyone related to you have a life-threatening illness, would you accept a stem cell treatment offered to you or recommend it?

- Yes
- No
- I do not know

14. Do you know that using unapproved stem cell treatment may cause serious complications and may result in having Cancer and/or other health complications?

- Yes
- No

15. Are you willing to travel to try a stem cell treatment or recommend it to a family member or friend?

- Yes
- No

16. Would you be interested in developing your knowledge about stem cells?

- Yes
- No
- I do not know
