# Supplementary material for: An Infodemic of Misinformation on Stem Cell Therapy Among the Population of Saudi Arabia: A Cross-Sectional Study
Source: Front Med (Lausanne). 2022 Mar 2;9:789695. doi: 10.3389/fmed.2022.789695 (PMC8924302; doi:10.3389/fmed.2022.789695)
Supplement: Supplementary file 2 [file Data_Sheet_2.docx]

**Supplementary Figures**

**Supplementary Figure 1.** **Objective knowledge of the participants**

1. Do you have a detailed knowledge of stem cells?


**b.** Do you have a general knowledge of stem cells?

**c.** Did you ever attend meetings regarding stem cells?

**Supplementary Figure 2.** **Participants’ overall interest towards developing stem cell knowledge in Saudi Arabia.**
